# Barriers and facilitators to dietary change while aiming to reduce free sugar intakes: framework analysis based on intervention, time for change, and success at 12 weeks

**DOI:** 10.1017/jns.2026.10118

**Published:** 2026-06-18

**Authors:** Lucy R. Boxall, Hannah Dalby, Emily Arden-Close, Janet James, Katherine M. Appleton

**Affiliations:** 1 Dept. of Psychology, Bournemouth University, UK; 2 Dept. of Nursing Science, Bournemouth University, UK

**Keywords:** Free sugar intakes, Knowledge, Motivation, Practical solutions, Qualitative

## Abstract

This study aimed to identify barriers and facilitators to reducing free sugar intakes while participants were in the process of attempting to do this. Sixty-two adults with free sugar intakes >5% total energy intake; participants in a randomised controlled trial examining the effects of three different dietary recommendations versus control for reducing free sugar intakes were interviewed at either 1, 2, 4, 8, or 12 weeks after receiving their recommendation. Data were analysed using thematic analysis and framework analysis based on recommendation received, time for change, and success in reducing free sugar intakes at 12 weeks. Thematic analysis revealed seven interactive themes leading to dietary change: ‘Is it possible?’; ‘Power of knowledge’; ‘Personal balance and empowerment’; ‘Habitual approach’; ‘Realities of life’; ‘Extensive awareness and viewpoint’; and ‘Proof and impact’. Framework analysis revealed greater knowledge, including knowledge related to practical solutions, in intervention groups compared to control; greater intentions and expectations at the start of the process, followed by increasing or decreasing engagement and satisfaction over time, with noticeable physical and subjective proof occurring from 4 weeks; and active engagement, with growing confidence and motivation in those who ended the trial having reduced their free sugar intakes compared to more passive attitudes in those less successful. Our findings demonstrate clear variation in barriers and facilitators to reducing free sugar intakes throughout the process, dependent also on recommendation received and individual orientation. Suggestions for improving free sugar intake reduction can be offered based on these differences.

## Introduction

High free sugar intakes are a major public health concern, associated with adverse physical health, morbidity, and mortality.^(1,2)^ Free sugar intakes remain above recommended levels for all ages around the world.^(3)^ In the UK, free sugar intakes exceed UK <5% total energy intake (TEI) guidelines,^(4)^ while countries like Australia, Austria, Germany, Netherlands, and Switzerland also consume over the <10% TEI guidelines of the WHO.^(3,4)^

To reduce free sugar intakes, it is imperative to identify effective interventions. Reviews predominantly on sugar-sweetened beverage consumption demonstrate some reductions following increases in price or taxes, changes to the food environment resulting in reduced availability of high-sugar items or increased provision of alternatives, and education to discourage intakes.^(5–9)^ Findings from these reviews, however, are highly heterogeneous, and interventions were found to impact different individuals to differing degrees.

Arguably, intervention success will depend on the determinants of the behaviour an intervention is trying to change and the degree to which individuals adhere to the intervention.^(10)^ A recent qualitative systematic review reported three types of barriers and facilitators to adherence to interventions for dietary behaviours.^(11)^ Individual factors were ‘Attitudes’, ‘Concern for health’ and ‘Physical changes’, referring respectively to individual opinions of and attitudes towards the behavioural guidelines, concern for current and future health, and positive or negative changes or lack of changes resulting from adherence. Environmental factors were ‘Social support’, ‘Social accountability’, and ‘Changeable community aspects’, and related to support from others, accountability for maintaining behavioural adherence to the self, to others or to society by maintaining social norms, and to community aspects such as the built environment (e.g. number of local food outlets). Intervention-specific factors were ‘Design and delivery’, ‘Content’, and ‘Fostering-self regulation’. These themes covered both the positive and negative options of information delivery and the characteristics of interventions that encourage or discourage engagement.^(11)^

In relation to reducing free sugar intakes, many of these individual and environmental factors have been reported to impact sugar consumption and proposed sugar reduction.^(12–16)^ Qualitative work by Rawahi et al.^(14)^ and Tang et al.^(16)^ finds reference to attitudes, heath concern, social and environmental factors in relation to sugar consumption, and associations between attitudes and consumption were subsequently demonstrated in a large cross-sectional study.^(15)^ In the review by Mazarello et al.,^(13)^ individual liking, the attitudes and practices of parents and significant others, and environmental factors, such as availability, were associated with sugar-sweetened beverage consumption in children,^(13)^ and in the review by Gupta et al.,^(12)^ individual liking, the attitudes of peers and significant others, and environmental cues were associated with attitudes towards sugar, although associations with consumption were weak.^(12)^ All studies also report influence from other determinants, most notably dietary knowledge or knowledge of current recommendations.^(12–16)^

Similar factors were also found when discussing proposed sugar reduction in the two qualitative studies^(14,16)^ and are considered in the review by Gupta et al.^(12)^ No studies of which we are aware, however, have investigated the barriers and facilitators to reducing free sugar intakes as part of an intervention in individuals undertaking this activity. To provide more effective interventions, assessing these aspects within their context of use is vital. This research aimed to explore barriers and facilitators to adhering to a free sugar reduction intervention across a 12-week period. The work was conducted through interviews with volunteers given one of four different interventions, who were interviewed at one of five different time points over the 12 weeks. Analyses were conducted by intervention group, interview time point, and degree of success in reducing free sugar intakes for added insight.

## Methods

The work presented here was undertaken within a randomised controlled parallel-group trial.^(17,18)^ In this trial, 242 adults (214 female, 18–65 years) were randomised to receive recommendations for reducing their free sugar intakes to less than 5% TEI as per UK government advice, which were nutrient-based (Group *N*) (*n* = 61), nutrient-and food-based (Group NF) (*n* = 60), or nutrient-, food-and food-substitution-based (Group NFS) (*n* = 63). A control group (CG) (*n* = 58) received no recommendations but were asked to accurately log food and beverage intakes, as were all intervention groups. All participants received their dietary recommendations at a single time point, in the form of an information booklet to take away, and outcomes were assessed for the following 12 weeks. Our primary outcomes were free sugar intakes as a percentage of TEI and adherence to the dietary recommendations, defined as a 2% TEI reduction in free sugar intakes from baseline or reaching the 5% TEI UK government guideline. Secondary outcomes included assessments of a range of dietary, anthropometric, and taste outcomes over the 12-week period, with qualitative interviews undertaken to investigate barriers and facilitators to intervention success. Full details of the trial methodology, primary, and the majority of secondary outcomes are reported elsewhere.^(17,18)^ This paper presents the qualitative methodology and findings.

### Ethical considerations

Ethical approval for the trial was given on 28.04.20 (with amendments approved on 29.03.21) from the Research Ethics Committee of Bournemouth University, UK (ref ID: 30612). The study was also registered as a clinical trial on Clinicaltrials.gov (ID: NCT04816955) on 24.03.21. All research was carried out in compliance with the Research Ethics Code of Practice of Bournemouth University, the British Psychological Society, and the Declaration of Helsinki (1983). Before taking part, all participants received a participant information sheet and provided written informed consent.

### Design

To allow comprehensive investigation of the barriers and facilitators involved in reducing free sugar intakes, interviews were undertaken with participants in all trial arms and at the end of weeks 1, 2, 4, 8 or 12 of the 12-week study period.

### Sample selection

To allow sufficient interviews from all four trial arms at each of the five time points, 60–80 interviews (3–4 per time point per trial arm) were originally planned. To ensure similar sample sizes in each intervention arm and at each time point, covariate adaptive randomisation was used.^(19)^ Once participants had been randomised into the trial and provided additional consent to undertake the interview, they were immediately assigned a provisional interview time point. Interview time point randomisation was carried out by a secondary researcher (KMA) with the main researcher interacting with participants (LRB) blinded to group allocation during all data collection. Each participant was allocated to only one time point and interviewed only once.

### Interviews

A semi-structured interview guide was developed specifically for this study. This guide asked participants about the barriers and facilitators they faced when following their dietary recommendation, with questions developed to cover: opinions/beliefs; feelings; knowledge; behaviour/experiences; sensory; and background or demographic characteristics. Questions were open-ended to encourage descriptive expression,^(20)^ with questions on opinions, beliefs, and feelings asked earlier to prevent disinterest.^(21)^ The order and wording of the script is included in **Supplement S1.**

### Data collection process

All interviews were undertaken via telephone or video-conferencing by the same researcher (LRB). Two methods of audio recording were used to minimise loss of data, and all audio recordings transcribed as soon as possible after each interview. All data were handled according to ethical guidelines, ensuring that once the call had taken place and the data transcribed, the original audio recording was destroyed. Only the anonymised transcription remained on a secure server.

### Data analysis

Data were analysed using framework analysis. Framework analysis is a comparative form of thematic analysis^(22)^ and was chosen as a suitable analytical method for evaluating our qualitative interview data, to allow comparison between intervention groups, across time points and based on degree of success.^(23)^ The method of framework analysis follows a previously published 7-phase methodology, based on thematic analysis,^(24)^ aided here with NVIVO software.

The transcribing author (LRB) developed the codebook in agreement with a second author (KMA) who doubled coded 10% transcripts for 100% agreement. All transcripts were then coded by LRB, and a further 40% of the transcripts were double coded between LRB and HD with 96.6% agreement. This resulted in an agreement of 97.3% across all transcripts and coders. Themes and subthemes were then generated by one researcher (LRB) and reviewed by two senior researchers (KMA and EAC).

Once themes and subthemes were agreed, one researcher (LRB) charted data onto a framework matrix with summaries created for each participants commentary across all themes and subthemes. A further set of summaries were created across group, interview time point, and degree of adherence to assess variation.^(25)^ For the purposes of these analyses, degree of adherence was scored at each interview time point (weeks 1, 2, 4, 8, 12) following completion of the whole trial, where participants were given a score of 1 if achieving ≤5% TEI from free sugars or ≥2% TEI reduction in free sugar intakes from baseline, or 0 if non-adherent, and all scores were summed, resulting in adherence scores per participant from 0 to 5. Connections and relationships between categories were then mapped with variations between groups, interview time points, and degree of adherence analysed. These connections and relationships were confirmed by a senior researcher (KMA) ahead of publication.

### Researchers and reflexivity

The main researcher (LRB) and secondary coders (KMA and HD) were all female, with a lean BMI. All three researchers had backgrounds in nutrition, psychology, and eating behaviour, with no history of mental or physical eating disorders or conditions. None of the researchers held strong opinions regarding current dietary trends and were not following any specific dietary programmes during the work or at the time of analyses.

## Results

### Participant characteristics

Sixty-two interviews (solo *n* = 59 and pair *n* = 3) were conducted, with a total of 65 individuals. Of the 59 solo interviews, 53 participants were female (F) and 6 were male (M). In the interviews conducted in pairs, participants resided in the same household and had received the same recommendation,^(18)^ with two interviews with couples (1M, 1F) (1F, 1F) and the third with a father and daughter (1M, 1F). The majority (59) participants were interviewed in 2021, with a final nine participants interviewed in the first quarter of 2022. Ten participants were aged 18–29 years, eleven were 30–39 years, twenty-four were 40–49 years and twenty were 50–65 years. The gender and age distribution of the interviewees reflected the participants in the whole trial.^(18)^ Interviews were conducted at all time points and with participants in all intervention groups (Table 1), with ranging degrees of success in reducing their free sugar intakes (Table 2).

**Table 1.** Interview frequency by group and interview week

	Control	N	NF	NFS
W: 1	2	1	4	2
W: 2	2	3	2	4
W: 4	3	1	4	5
W: 8	1	5	3	5
W: 12	3	4	3	8

Numbers shown are interview frequencies. Abbreviations: Week (W), Nutrient group (N), Nutrient and food group (NF), Nutrient, food, and food substitutions group (NFS). Control group (*n* = 11), *N* (*n* = 14), NF (*n* = 16), and NFS (*n* = 24). W1 (*n*=9), W2 (*n* = 11), W4 (*n* = 13), W8 (*n* = 14), W12 (*n* = 18).

**Table 2.** Interview frequency by group and adherence score Table 2 long description.

Adherence score	Control	N	NF	NFS
Zero	2	2	0	2
One	3	1	1	2
Two	3	2	3	4
Three	0	3	2	7
Four	2	1	3	3
Five	1	5	7	6

Numbers shown are interview frequencies per summative adherence score. Abbreviations: Nutrient group (N), Nutrient and food group (NF), Nutrient, food, and food substitutions group (NFS). Control (*n* = 11), *N* (*n* = 14), NF (*n* = 16), and NFS (*n* = 24), Adherence score: Zero (*n* = 6), One (*n* = 7), Two (*n* = 12), Three (*n* = 12), Four (*n* = 9), Five (*n* = 19). Adherence was scored at each time point by either achieving ≤5% TEI from free sugars, or ≥2% change in TEI from free sugars from baseline, higher scores denote greater adherence.

### Thematic analysis: barriers and facilitators to intervention adherence

**Figure 1.**
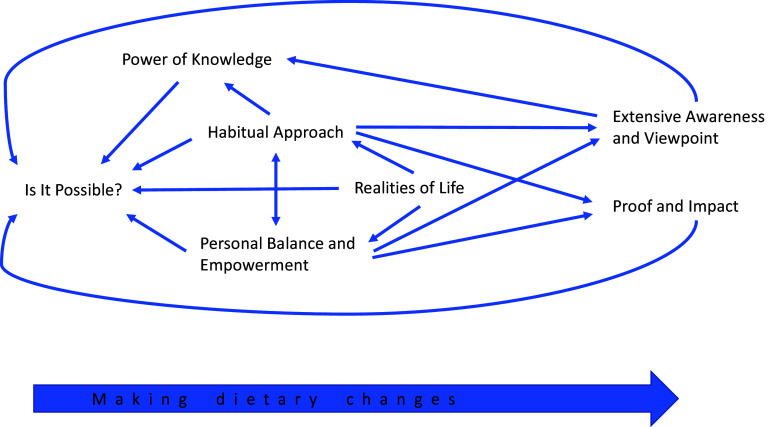
Figure 1 long description. Thematic map depicting the seven themes from the thematic analysis and their inter-relations.

Using thematic analysis, a total of seven themes and fourteen subthemes for barriers and facilitators to intervention adherence were identified and are described below. There was considerable interaction between the themes, and interaction also with dietary change, such that adherence to the recommendations increased further adherence, while failure also encouraged failure. All themes, their definitions and subthemes are given in Table 3. Themes and their inter-relations are also depicted in Figure 1.

**Table 3.** Themes, their definitions and subthemes Table 3 long description.

Theme	Definition	Subthemes (if applicable)
Is it possible?	*Are the recommendations achievable and what aspects were considered positive or negative regarding the advice provided.*	–
Power of knowledge	*Clarity of knowledge and the power of understanding, including the limitations of the absence of knowledge.*	Enables, Disables
Personal balance and empowerment	*Our personal approach to the world, attitudes, personality and thought processes can be positive or negative for intervention adherence/nonadherence.*	Balanced, Unbalanced, Empower, Disempower
Habitual approach	*Practical habits for adherence to recommendations and for maintaining any changes made.*	Active, Passive
Realities of life	*Our individual or societal environment and daily habits can facilitate or limit adherence to recommendations.*	Facilitators, Barriers
Extensive awareness and viewpoint	*Broadened awareness of food, and the influences of the government, family and friends on intakes.*	–
Proof and impact	*The impact and perceived proof gained from adhering to the intervention recommendations.*	Seeing proof, Limited impact

#### Is it possible?

*Are the recommendations achievable and what aspects were considered positive or negative regarding the advice provided.*

The theme ‘is it possible?’ conveys the reported achievability of the dietary recommendations provided for both the individual and for wider society. It refers specifically to the nature of the advice provided as well as participant perceptions of this. Participants generally regarded the recommendations as achievable, both for themselves and for others, and there was positivity and optimism, with recognition that certain factors, such as food labelling, may be helpful.

*I liked the d-dietary recommendation um, because it was very simple. (P29, Group N, Week 8)*

#### Power of knowledge

*Clarity of knowledge and the power of understanding, including the limitations of the absence of knowledge.*

The theme ‘power of knowledge’ describes the effect of having, or not having, knowledge, on undertaking and adhering to the recommendations. This theme is subsequently split into two subthemes: **‘Enables’** and **‘Disables’**. Factors that improve knowledge were considered to likely enable individuals to make informed choices and decisions.

*Urm, so it’s knowledge, so I’m sure if I didn’t have knowledge and someone gave me some of those favourite foods again, I might go “ooh this is nice” and be tempted again, but now that I know I definitely don’t think I’ll go back to most of them. (P31, Group N, Week 12)*

Education was considered especially important from a young age, with some suggestion that more could be done by the government. Commentary was provided on nutritional labelling, ingredient lists, the need for clarification on foods high in free sugars, and on understanding sweeteners and foods substitutes. Conversely, not having knowledge, confusion and misunderstanding, or being presented with conflicting advice were considered to result in more negative outcomes. Comments were provided on poor labelling, deception by the food industry leading to hidden ingredients and foods being advertised as healthier than they are, and with specific challenges highlighted when eating out.

*Um, I think it’s, it’s not always easy when you’re sort of bombarded with advice from you know all media types. (P57, Group NFS, Week 2)*

#### Personal balance and empowerment

*Our personal approach to the world, attitudes, personality, and thought processes can be positive or negative for intervention adherence/nonadherence.*

Separate from the realities of life (as below), this theme includes our attitudes, emotional responses, and personality and is split into the four subthemes of ‘**Balanced’, ‘Unbalanced’,’ ‘Empower’,** and **‘Disempower’**. The theme regards how we respond to challenges and change. Are we likely to be more positive, or negative? Have a balanced or unbalanced view of the world? For example, descriptions of foods such as ‘naughty’, ‘treat’ or referring to days as ‘bad days for eating’, or extreme thinking towards eating, that may be beneficial for a time, but not long term, were considered ‘unbalanced’. Conversely, having a ‘balanced’ view of dietary factors was described as eating in moderation across different food groups and differing eating occasions.

*So, it’s kind of just, it’s a balance, and it’s it’s about kind of, you know, not saying never to anything, because I think if you block out an entire food group, then actually that can cause more problems, so it’s about balance and moderation and just being aware. (P106, Group NF, Week 12)*

Participants also perceived themselves and others as either having free choice and responsibility to ‘empower’ their dietary intakes or not, with motivations identified for self, family, and/or health. The personality trait of having willpower was described as positive for adherence (empower), whereas the inverse, lacking willpower was described as having a negative effect on adherence (disempower). Some people described not being able to make changes due to willpower, self-discipline, just not being motivated to make the change, or from being strongly influenced by other people and other external factors, such as competing priorities or government restrictions (disempower). These situations were further associated with negative emotions resulting from eating foods participants knew they should not eat, while empowerment could come from making a specific food choice from a knowledge base, with positive emotions resulting from these subjectively ‘better’ choices.

*But I think ultimately it is that individual’s freedom of choice, urm and their responsibility, urm, it’s just that some people can be influenced more than others. (P9, Control Group, Week 1)*

#### Habitual approach

*Practical habits for adherence to recommendations and for maintaining any changes made.*

In this theme, participants described the changes they ‘had’ or ‘had not’ made regarding their eating behaviours and habits. The theme describes both the likelihood of change and the changes the individual undertook. Participants described avoiding certain foods, most noticeably snacks, having made swaps or food substitutions, or suggested reduced-sugar versions of regular foods. The subthemes **‘Active’** and **‘Passive’** describe how participants approached or described changes. For example, had there been an active approach to habitual change, for example, swapping snacks, increasing knowledge, increased meal-planning and home-cooking.

*I’ve been choosing fruit as a substitute for, instead of having a bar of chocolate. (P97, Group N, Week 12)*

Alternatively, the individual may have had a more passive approach, for example, relying on others or study requirements to maintain motivations for change, or they did not think they needed to or cared to change. Additional comments also regarded lifestyle changes such as getting back to exercise, going to the gym now, or expanding their health knowledge.

*Um I think I’m good on the days where you’re asking us to record our food. ((both laugh)) (P64 and P65, Group NFS, Week 2)*

#### Realities of life

*Our individual or societal environment and daily habits can facilitate or limit adherence to recommendations.*

Individuals described personal and wider environmental factors and daily habits that did, or could, affect their adherence to the dietary recommendations they were given or to a diet they perceived to be healthier. This theme is split into the two subthemes of **‘Facilitators’** and **‘Barriers’**. The subtheme **‘Facilitators’** outlines instances that individuals described as being helpful for dietary change, for example, healthy food access and motivation. Planning was described as helpful for better food choices, alongside if healthier food was cheaper and if friends and family were supportive, or could influence participants to make healthier decisions. Living alone was also mentioned to be helpful as it made participants more able to control the foods they bought and consumed, including regulating portion sizes.

*I’ve hopefully got a bit more time, err, to do that, urm, and a bit more motivation with some goals in mind. (P16, Group N, Week 2).*

The subtheme ‘**Barriers’** outlines instances that participants described as being unhelpful for adherence to dietary advice, such as being busy at work, not having enough time, eating away from home or travelling. Junk food was perceived as being cheaper, with healthy food described as often being more expensive or less accessible. Comments also regarded other people’s eating habits within the household and difficulties in incorporating the views of others into eating practices and occasions.

*Urm, because society doesn’t agree with what your recommendation was. So, so sort of going against what society is doing if that makes sense.(P31, Group N, Week 12)*

#### Extensive awareness and viewpoint

*Broadened awareness of food and the influences of the government, family, and friends on intakes.*

This theme describes participants views on the impacts of the study on their awareness of different food items, their food intakes and of outside influences on food intakes. Participants provided descriptions of being more conscious of eating; both individuals and the government were suggested to have responsibility for food, dietary intakes, and enabling healthy choices, while the food industry was also given some responsibility for providing healthy foods, not adding unnecessary ingredients to foods, and providing transparent labelling. Extensive awareness stemmed from participating in the research study, both via the recommendations provided and the act of dietary recording, but certain preconceptions were also considered to be present before participation and may also have influenced subsequent perceptions and interpretations.

*Um, but I think um, just realising actually w-what content was in certain foods…there was a lot more affected, er, or a lot more foods had more impact. (P37, Group NFS, Week 12)*

#### Proof and impact

*The impact and perceived proof gained from adhering to the intervention recommendations.*

This proof and impact appeared from the conscious and/or subconscious searching for proof of their adherence to the dietary recommendations they had been given. Participants commented on changes they had or had not seen during their participation, and this included changes to their diets, with dietary recording useful for seeing an overview of the diet and for making food decisions. This theme is split into two subthemes of **‘Seeing proof’** and **‘Limited impact’**. Seeing proof included positive or negative proof of impact for their efforts, such as experiencing any physical, subjective or dietary changes, including changes to the tastes of foods.

*So I think you know, like the changes that I’ve made with the couple of food items that I’ve changed, I think that you know, that does make you feel better, um that you found something that actually fits with the way that you want to eat and you know is healthier, um, so that’s a really nice change, um, and really positive from this study. (P57, Group NFS, Week 2)*

Limited impact regarded individuals not seeing any changes, with their participation in the study or with the recommendations having no impact on their life, for example, no changes to eating behaviours, food choice or dietary knowledge.

*Urm, no, other than just finding it a hassle to follow the recommendations that I was given. Other than that, it hasn’t made any change to my life. (P15, Control Group, Week 12)*

### Framework analysis: comparisons by group, interview time point, and degree of success

#### Comparison by group

An average of 16 interviews were completed in each group: Control group (*n* = 11), Group N (*n* = 14), Group NF (*n* = 16), and Group NFS (*n* = 24). Overall, commentary increased across the groups in this order, providing increasing description and detail. Thus, participants in the NFS group tended to contribute the most. Responses from the control group (CG) tended to cover similar topics as those of the intervention groups (IV); however, these responses were more general and less personalised. The full comparison between themes is shown in Table 4.

**Table 4.** Group comparison results Table 4 long description.

Theme	Similarities	Differences
Is it possible?	Recommendations are achievable, with dietary recording helpful.	CG – consistent opinions that action was straightforward. IV – knowledge of baseline intakes would have been helpful for change. Recommendation achievability was considered more difficult at the very start or over the long term.
Power of knowledge	Importance of nutritional education, with acknowledgement of youth and government contexts. The government could do more (enables). Poor labelling of multipack and baked goods, or poor ingredient descriptions by industry (disables).	CG – lack of information is challenging. IV – substantial commentary on nutritional labelling, for example, traffic light system; some foods advertised as healthier than they are; challenges eating out when following guidelines. N – lack of information is challenging (disables). NF – clarification on foods high in free sugars, clarification on understanding sweeteners and food swaps (enables). NFS – clarification on foods high in free sugars (enables); information sources can be conflicting (disables).
Personal balance and empowerment	Foods referred to as ‘bad’ or ‘treats’ (unbalanced). Food groups eaten need to be balanced (balanced). Motivations were self, family or health. Individuals have free choice and require strong willpower to resist cravings (empower). Some individuals are more easily influenced by others, and/or unable to make changes due to low self-discipline, motivation or willpower (disempower).	CG – focused on balance between intakes and exercise (balanced). IV – empowerment in food choices due to knowledge, positive emotions from subjectively viewing new choices as better (empower); artificial ingredients, for example, sweeteners were not preferred or avoided (unbalanced). NF/NFS – compensating for eating habits over multiple days (empower); impacts of social situations and not wanting to pressure others with dietary preferences (disempower). NFS – provided specific circumstances for using substitutions and more personalised eating based on bodily symptoms and cravings (empower).
Habitual approach	Being accountable, but also recognising the influence of external observation on intakes (passive). Diet changes focused on snacks (active).	CG – few specific changes, with a greater focus on dietary planning and healthy eating in general (active). IV – dietary changes specific to certain foods or having made substitutions (active).
Realities of life	Eating away from home and travelling given as barriers due to difficulties in sourcing healthier choices. Negative influences of nefariously perceived food industry motivations and drive on free sugars. Time management, busy lifestyles, lack of knowledge as barriers. Junk food is cheaper, healthier food is less accessible (barriers). Planning and greater price accessibility for healthy foods. Supportive and similar mindset from family and friends (facilitators).	IV – gradual changes, physical distance from certain foods, living on your own, all mentioned as facilitators. Barriers included household not adopting similar dietary changes. There was more commentary on controlling food advertising and how the focus is often on unhealthy foods (barriers). NF/NFS – most descriptive, more reference to personal facilitators, for example, moderated eating and portion size control (facilitators), negative emotions from rejecting food offers from family/friends (barriers).
Extensive awareness and viewpoint	More conscious of eating. Individual and government have responsibility for intakes. Industry adds unnecessary ingredients to foods.	CG – comments were more generalised. IV – contributed more commentary on food labelling, with all comments communicated at a more personal level.
Proof and impact	Dietary recording is useful (seeing proof). Comments were uniform when referring to a lack of impact (limited impact).	CG – provided low contribution to ‘seeing proof’ NF/NFS – commented on physical changes, for example, improved energy, weight loss and general wellness (seeing proof).

CG, Control group; IV, Intervention groups; N, Nutrient group; NF, Nutrient and food group; NFS, Nutrient, food, and food substitutions group.

Where comments refer to subthemes, the subtheme is shown in parentheses.

#### Comparison by interview time point

The following numbers of individuals were interviewed at each time point, week one (*n* = 9), week two (*n* = 11), week four (*n* = 13), week eight (*n* = 14), and week twelve (*n* = 18). Similarities and differences between time points are given in Table 5.

**Table 5.** Interview week comparison results Table 5 long description.

Theme	Similarities	Differences
Is it possible?	Recommendations generally described as achievable.	Earlier interviews – individuals ‘trying’ to achieve guidelines Weeks 8/12 – descriptions of fatigue with dietary recording, and uncertainty whether changes made were correct.
Power of knowledge	Individuals regarded themselves to already having good knowledge (enables), with labelling described as deceptive by the food industry (disables).	Weeks 8/12 – food labelling described as being poor with criticisms of nutritional information, portion sizes, traffic light ingredients, and jargon (disables). Week 12 – individuals describe making more informed choices and knowing where to find information (enables).
Personal balance and empowerment	Recognition of personal responsibility and willpower for eating (empower). Healthier choices participants can make (balanced). Not choosing foods because they are artificial and avoidance of these (e.g. sweeteners) (unbalanced).	Later interviews – awareness of bodily needs and craving, recognition of the value of diet personalisation (balanced). Discussions become more enlightened around food packaging and what they had learnt (empower). Weeks 8/12 – more comment on change in treat style (unbalanced).
Habitual approach	Observation and/or study participation provided motivation to make changes (passive).	Earlier weeks – changes back to fresh cooking (active), Week 2 onwards – food variety increased, specifically fruit and veg (active). Later interviews – changes made, or sticking with them (active). Weeks 8/12 – habits described as harder to change, instances of individuals set in their ways (passive).
Realities of life	Reducing portion sizes but not ‘cutting out’ (facilitators). Reporting of lifestyle barriers were similar across interview weeks with no discernible differences (barriers).	Earlier weeks – how other people can facilitate changes (facilitators). Later interviews – friends and family as facilitators when having similar outlooks on diet (facilitators).
Extensive awareness and viewpoint	N/A	Weeks 1/2 – participants are thinking about changes. Weeks 2/4/8 – increased awareness on what participants eat linked to dietary recording. Later interviews – focus on labels and nutritional information. Week 12 – comments on changes that had been made.
Proof and impact	Dietary recording is useful. Physical and taste changes reported from week 1 (seeing proof).	Week 4 onwards – comments on increased energy, and physical changes such as improved skin and weight loss (seeing proof).

*Footnotes:* Where comments refer to subthemes, the subtheme is shown in parentheses at the end of each section.

#### Comparison by degree of success in reducing free sugar intakes

Individuals had the following adherence scores by the end of the study (not necessarily at the time of the interview): Zero (*n* = 6), One (*n* = 7), Two (*n* = 12), Three (*n* = 12), Four (*n* = 9), and Five (*n* = 19). Similarities and differences between comments based on degree of success are given in Table 6.

**Table 6.** Adherence score comparison results Table 6 long description.

Theme	Similarities	Differences
Is it possible?	Recommendations generally thought of as achievable.	Lower adherence scores – confusion regarding recommendation boundaries; focused on ‘trying’ to adhere. Higher adherence scores – confidence that they were following the advice.
Power of knowledge	Enables and disables subtheme responses were similar.	Higher adherence scores – commentary on food specifics such as clarification on not understanding what’s best for us; whether information is disingenuous; and whether information from the government is actually what is right for us or just what they thought was achievable.
Personal balance and empowerment	N/A	Lower adherence scores – reports of responsibility not just on the individual, and some rebelliousness towards ‘nanny state’, which individuals commented could cause rejection of recommendations (disempower). Higher adherence scores – recognition of the relationship between physical effects and dietary decisions (balanced). Adherence scores of two and above – health conditions and emotional eating impacts discussed (balanced). Adherence scores of five – how some people will never change (unbalanced), also incorporated more descriptive responses including energy intakes, balanced diets and lifestyle (balanced).
Habitual approach	Being better on days of dietary recording, by making participants more accountable for their intakes (passive).	Adherence scores of three or more – additional comments regarding changes such as getting back to the exercise they were doing, going to the gym now, or expanding their knowledge (active).
Realities of life	If healthier food was cheaper and if ‘bad’ foods had limited proximity, were facilitators. Price, access, going out to eat, socialising, were barriers.	Adherence scores of two and above – described barriers such as the influence of food advertising and how the industry could be misleading and employ deceptive marketing (barriers).
Extensive awareness and viewpoint	Increased awareness originated from food labelling; knowledge expansion and dietary recording.	N/A
Proof and impact	Comments on limited impact had no discernible differences. Taste changes reported, with individuals enjoying foods they had altered intakes for, for example, fresh fruit and savoury foods (seeing proof).	Adherence scores of two or more – physical changes such as having more energy, clearer skin, and weight loss were more commonly mentioned (seeing proof).

*Footnotes:* Where comments refer to subthemes, the subtheme is shown in parentheses at the end of each section.

## Discussion

The framework analysis completed in this study elucidates the experiences of participants when asked to follow a dietary recommendation aiming to reduce free sugar intakes. Thematic analysis first identified seven themes with fourteen subthemes, and framework analysis was subsequently used to identify differing experiences, barriers and facilitators to dietary change based on recommendation type, time since recommendation provision, and degree of success.

### Thematic analysis

The seven themes from the thematic analysis (Is it possible?, Power of knowledge, Personal balance and empowerment, Habitual approach, Realities of life, Extensive awareness and viewpoint, Proof and impact) reflected what people thought of the change to their diet that they were asked to make, what people did, and their reflections on both of these aspects. All themes included elements that both facilitated dietary change and elements that acted as barriers. On the cognitive side, the theme ‘Is it possible?’ describes participants perceptions of the feasibility of the request. This was closely linked to the ‘power of knowledge’ theme, which demonstrates clear recognition of the importance of knowledge or lack of knowledge for dietary change. Both perceived feasibility of dietary change and increased nutritional knowledge have previously been identified as important in following a healthy diet,^(26–28)^ with the provision of educational materials resulting in improvements.^(29–31)^ Gupta et al.,^(12)^ Rawahi et al.,^(14)^ and Tang et al.,^(16)^ also mention knowledge or lack of knowledge, specifically in association with sugar intakes, and increased knowledge has been suggested to aid with reducing, increased motivation to and likelihood of changing, sugar intakes.^(12,14,16,32,33)^ While some of our participants will have already had some dietary knowledge on study entry, it is likely that almost all benefited from the knowledge provided.

Perceptions of the intervention were also closely tied to participants thinking style, preconceptions, and understanding of the world around them, as reflected in the themes ‘personal balance and empowerment’ and ‘habitual approach’. Of these, the theme ‘personal balance and empowerment’ can be considered an individual’s attitudes towards life in general, to include personal strength and perceptions of feeling empowered, capable and in control, or not. The theme ‘habitual approach’ reflects more the participants’ attitudes towards challenges or prospective changes. This theme describes both active or passive engagement with the challenge and describes some of the actions that participants undertook, or not. Underlying ideas of self-efficacy, self-control, and a belief in oneself and one’s abilities^(34,35)^ link these two themes, resulting in attitudes and practices that are broadly either positive or negative, and broadly either supportive or not supportive of enacting health behaviours such as eating healthier food and exercising more.^(34–42)^

Attitudes that are broadly positive or negative, and supportive or not, will also feed into the above mentioned themes. As an example, the *desire for knowledge*, alongside activities *to gain knowledge* or *an active engagement with knowledge*, as part of a ‘habitual approach’, as well as knowledge itself, have been found to be important for intervention adherence, and negative attitudes towards guidance have been found to be a hindrance.^(11)^ Thus, individuals with a positive sense of ‘personal balance and empowerment’ and an active ‘habitual approach’, on receiving their recommendation, may have undertaken additional research or investigation into aspects of the recommendations, such as the free sugar content of foods. This additional information may then enhance knowledge as expressed in the theme ‘power of knowledge’, with this further knowledge also potentially contributing to perceptions of the achievability of the recommendations in the theme ‘Is it possible?’.

Linked to the theme ‘habitual approach’, the theme ‘realities of life’ describes wider or more established influences on diet, some of which may facilitate change and some of which may hinder it. It is widely acknowledged that our social and physical environment impacts dietary factors such as the availability, convenience, and acceptability of food,^(43–45)^ with impacts on dietary intakes and likely adherence to dietary recommendations.^(46,47)^ Impacts of these factors specifically for sugar intakes are also recognised.^(12-14,16)^ Importantly, these factors were largely considered outside of the control or purposive control of the individual. Thus, the themes ‘personal balance and empowerment’ and ‘habitual approach’ reflect the individual’s role in what happens, while the ‘realities of life’ theme reflects more what happens to them.

The co-existence of active and passive perspectives was also found in the final themes ‘extensive awareness and viewpoint’ and ‘proof and impact’. The theme ‘extensive awareness and viewpoint’ describes gains in knowledge and recognition of the influences of outside forces in dietary change. Investigations into dietary recording have consistently shown that the recording of dietary intakes improves factors such as nutritional knowledge, food choice, portion size, and awareness of diet–disease relationships.^(48–50)^ The potential impact of this ‘increased awareness’ on physical outcomes has also been described, with individuals in weight-loss studies who recorded diet diaries observed to lose considerably more weight than those who did not.^(49–51)^

The theme ‘proof and impact’ recognises the importance of physical and other changes that can be perceived by the individual. This evidence is reported as important for remaining in weight loss interventions^(49,52–57)^ and arguably may apply to all dietary change settings. This theme suggests a need for evidence of impact for some individuals, more so than that of increased awareness, linking to elements of the ‘personal balance and empowerment’ and ‘habitual approach’ themes. The theme ‘extensive awareness and viewpoint’ also linked to the ‘power of knowledge’ theme, and in turn to the theme ‘Is it possible?’. The interconnected nature of the themes, and their underlying interdependence on attitudes and perspectives that can be broadly considered as ‘active’ or ‘passive’ is of interest.

### Framework analysis

Considering the relevance of the changes people were asked to make, their reflections on these and on the changes they made, differences may be expected between groups, over time, and based on the degree of success they achieved. Similarities across all three factors included that: the recommendations were generally considered achievable; knowledge and dietary recording were considered helpful; choice, moderation, motivation, willpower, accountability both towards themselves and towards the study, and individual responsibility were beneficial for healthy changes; with some social and physical environments, including large-scale concerns, such as government directives and the food industry, considered supportive and others acting as barriers to change. Benefits were described from increasing dietary knowledge and awareness, from making small changes, for example, reducing portion sizes, and from seeing proof of any changes made, either in the form of healthier diets or in terms of physical health and subjective experiences. All factors have previously been reported when attempting and achieving dietary change, both as facilitators of change and with the reverse of these factors as barriers.^(11)^ Similarities within groups, over time, and based on degree of success are also unsurprising, given that all individuals were asked to undertake a dietary change and had knowingly volunteered for this as part of a study of diet and behaviour, during which their diet and behaviour were monitored. Differences, however, between groups, over time, and based on degree of success, were also found.

#### Group comparison

Regarding differences between the groups, the recommendations for the control group were perceived as straightforward and general, resulting in general discussions on healthy eating, healthy lifestyles, and little evidence of impact. Participants in the intervention groups provided more informed discussion on the feasibility of the changes required with reference to the value of knowledge, with recognition also that a lack of knowledge or conflicting knowledge can be detrimental. The more detailed discussion likely reflects the increased information provided as part of the interventions. The value of knowledge was further specified in relation to food ingredients, certain foods, and food substitutions, resulting in healthy and empowering food choices. Lack of clarity on existing intakes, and a lack of information on food labels, packaging, or when eating out were highlighted. This value of knowledge for facilitating dietary change is well-known, as mentioned above.^(12,14,16,26–28,32,33,42)^ The specific knowledge referenced reflects the information provided as part of the intervention and demonstrates the value of the differing information provided on nutrients, foods, and food substitutions. Our quantitative results also suggest value to the differing information provided across our recommendations,^(18)^ not only for reducing free sugar intakes, but also by providing the different information needed by different individuals, allowing individual flexibility and choice.^(18)^ Free choice and individual autonomy are recommended for health behaviour change, thus allowing for individual differences in preferences, circumstances and priorities.^(55,57–61)^ The information requested on existing intakes and the difficulties found with food labelling and when eating out are interesting points of feedback for future use.

Detail was also found, particularly in the commentary from NF and NFS groups suggesting benefit from small, gradual and specific changes, such as moderated eating, portion size control, and specific food swaps. The benefits of small dietary changes, for sustaining behaviour change over time have previously been reported, with benefits for health.^(62–66)^ The specific changes mentioned likely reflect the specificity of our behavioural goal for these groups (to reduce free sugar intakes) and demonstrate value for this specificity and clarity in the dietary goal. Any behaviour change can be facilitated through the use of a clear goal,^(10,49,67–69)^ and the predominance of these comments by participants in NF and NFS groups suggest value to the more practical information provided for these groups in the form of food reductions and substitutions, compared with the more abstract nutrient-based detail. Other studies have successfully reduced free sugar intakes through the use of food substitutions or instructions to do this.^(70–72)^ This benefit of reference to foods rather than nutrients is furthermore recognised in the current push for food-based dietary guidelines.^(73)^

Positive impacts from changes were also more often reported by intervention groups compared to control, particularly in NF and NFS groups, and these visible changes likely reinforced behaviours and acted as facilitators to further change. This, combined with a likely increase in knowledge for the intervention groups, may have further enhanced motivation and adherence to the intervention.^(42,49,50,55,57–61,74)^ Increased recognition of potential barriers such as inadequate food labelling and resistance to change from family members was also more apparent in intervention groups compared to control. This increased recognition of these potential barriers may also be linked to increases in knowledge, where increased knowledge may allow increased recognition of existing barriers, such that these may be brought to the forefront.

#### Time-point comparison

Differences over time were also found. In early weeks (weeks 1 and 2), participants were ‘trying’ to make changes and looking towards the changes they could make or strategies they could use, such as increased home-cooking, and looking towards sources of support. This looking forward reflects the expectations individuals often engage with at the start of a behaviour change for which they have volunteered, and with which they are engaged and committed.^(49,57,74)^ The screening process to enter the trial was deliberately designed as a test of commitment and engagement to inform participants and reduce drop-out due to unexpected participant burden. The provision of strategies for implementation, however, also suggest active engagement with real intentions to change.

In later interviews (from week 4 onwards), participants reflected on the changes they had made or the effects they had noticed, in their diet and/or in their physical health, with uncertainty in the changes they had made if these effects were not as expected. Reports of taste alterations in response to whole-diet dietary interventions have been found,^(72,75)^ as have physical changes, for example, changes in body weight and shape,^(52–54,56)^ and changes in subjective experiences and perceptions, such as in energy levels, self-esteem, well-being, and body image.^(55,57)^ The need for proof or some evidence of benefit from the efforts undertaken is commonly reported in participants of health interventions and can be key to sustained motivation and implementation, as mentioned above. The timing of effects, in this regard, is of interest. Changes to dietary intakes occurred from the start of the intervention, leading to significant improvements in diet.^(18)^ Changes in food taste were also noticed after one week, and other studies also find changes to taste after short periods of dietary change.^(75)^ Physical changes, for example, in energy levels or body weight, however, were not noticed for a further three weeks. Other studies also recognise the time lapse required for participants to notice physical changes in response to lifestyle change and recognise the demotivation that these delays can cause.^(11,49,55,57)^ Much of the drop-out from dietary and lifestyle interventions occurs prior to this time point and often as a result of perceived failure. Our findings suggest that individuals need to be advised that changes may be unlikely before this time, and that additional aid and encouragement may be important for continued engagement in the initial phase. Beyond four weeks, however, external aid may be less important, as observed effects will start resulting in improved engagement and motivation.

Participants also described increased knowledge and active engagement with all available information in later weeks, resulting in considerable dissatisfaction with nutritional labels and the nutritional information provided on food packaging and increased awareness of their intakes. Ongoing increased knowledge is reported following information-based interventions,^(29–31)^ but the dissatisfaction with nutrition labels and the information on food packaging was a particular feature of this study. Links between nutritional knowledge and label use have previously been reported,^(76)^ and this comment is particularly understandable in relation to free sugar intakes in the UK, as many food product labels in the UK do not provide information on free sugars specifically. Labels are more often written using ‘Of which, sugars’ and ‘Total sugars’.^(77,78)^ The challenge of nutritional disinformation from the media, internet, and food industry has also been noted as a health communication challenge, with not all individuals having the health literacy to be able to critically evaluate all available information.^(27)^ These reports reflect the wider environment in which participants find themselves, highlighting the need for more consistency to be given between dietary guidelines and food labelling, so as not to disadvantage consumers. When the next review of UK dietary guidelines and/or food package labelling occurs, this process should be integrated to better consider both factors. This could then produce labelling formats that directly align with government dietary guidance.

The value of increasing awareness of dietary intakes for dietary change is well recognised^(49–51)^ and in fact warranted our use of a control group who only recorded their diet to control for the effects of this activity. The use of self-monitoring and feedback is recommended for maintaining positive behavioural changes,^(49–51,79)^ such that positive changes are supported, and ineffectual or incorrect changes can be corrected.^(80)^ Fatigue with dietary recording however is also well-known,^(81,82)^ and it has been suggested that negative effects on eating and the thinking around eating can arise following extensive dietary monitoring, although effects may be limited to certain individuals.^(83,84)^ There was some evidence of increasingly restrictive thinking over time in our study, for example, through the description of ‘good’ and ‘bad’ foods, or changes to the use of ‘treats’. These changes may be less desirable and demonstrate the need for care and nuance when designing dietary recommendations.

#### Comparison based on degree of success

Differences between individuals were also noted based on degree of success. First, those who ended the trial having been less successful in changing their diet reported greater confusion over the recommendation they received, and some distrust of the value of the recommendation and of the government in general. They provided comments around ‘trying’ rather than taking active steps and abdicated at least some personal responsibility for their diet and health. Those who ended more successful reported greater confidence in their abilities, more specific knowledge, and demonstrated active consideration of the knowledge they gained, discussing both helpful elements and distrusted or deceptive elements. The importance of knowledge and confidence in abilities or self-efficacy, for healthy diets and dietary change are discussed above. Links between these concepts are also obvious considering success. Active engagement with the information provided not only expanded knowledge, but also resulted in increased self-efficacy, motivation to change, and/or potentially viewing changes as easier to make.^(11,49,50,55,57–61,74)^ The act of doing further research may even demonstrate an element of personal investment,^(11,58,59)^ resulting in further motivation and success.

The issue of responsibility, where increased acceptance of personal responsibility is associated with more healthy diets and more health practices in general, is of interest. Research suggests positive associations between taking responsibility for one’s health and increased health behaviours when personal responsibility is aligned with perceptions of control, self-efficacy, and autonomy,^(40,85)^ although negative impacts can also arise if increased personal responsibility is associated with increased blame and stigmatisation for poor health behaviours or outcomes.^(85,86)^.These findings have resulted in previous suggestions for a greater preferred focus on increasing perceptions of self-control, self-efficacy, and autonomy rather than on personal responsibility as such.^(40)^

Those who ended the trial more successful also recognised the role of dietary decisions, not just for diet but for healthy lifestyles. They provided comments on physical activity and other health behaviours and discussed more evidence of making healthy dietary changes in physical and subjective experiences. Changes in visible bodily features, for example, weight loss, or subjective experiences such as increased energy were reported as facilitators, likely to impact adherence, with those ‘seeing proof’ more likely to continue with changes, whereas those reporting ‘limited impact’ were likely demotivated and more likely to give up.

### Recommendations for dietary interventions

Reflecting on the analyses presented above, there appear to be some key aspects relevant for use in future dietary interventions aiming to reduce free sugar intakes. Providing knowledge and enhancing understanding would appear to be key. Information should be provided in a form that is accessible to a wide population, and added value may be gained from confirming understanding by those concerned. Of particular note, in relation to free sugars, difficulties were expressed with the lack of information on food packaging, the use of jargon in food labels, and the misinformation perpetuated by the media. Clear and specific instructions were welcomed. Practical information was particularly valued by those receiving it in the form of the foods to target and possible food substitutions or alternatives. Participants also welcomed suggestions for small changes, such as a reduction in portion sizes, or use of similar low-free-sugar products.

Particular benefit may also be gained from personalised information, tailored to individual preferences and motives, and personalised feedback on the necessary changes, on successes and further requirements may also help. While this type of information provision may not be possible in traditional public health interventions, digital interventions can now provide this level of feedback, can offer personal choice, and may demonstrate superior changes.

Related to knowledge, practical solutions, and personalisation, likely benefits can also be suggested from increasing confidence, perceptions of control and increasing motivation. Active participation in an intervention will increase confidence and motivation, and accountability both to the self and to external parties appears to be facilitative. While accountability to external parties appears beneficial however in early stages, theory would suggest increased benefit from a gradual move towards greater self-interest and self-reliance.^(58–61)^ Adherence in early stages may also be aided by highlighting the likely unseen nature of any benefits, and any incidental changes, such as new friendships. What was important from our study was that the four-week time point appeared particularly poignant for participants. At four weeks, there was increased reporting of observable physical and subjective changes, with this a pivotal point impacting motivation and therefore sustained behaviour change. Also important was the observation, both by ourselves and by our participants, that success tended to lead to further success, while failure tended to lead to further failure. Initial engagement, commitment and action, then are important, to result in early success, ongoing engagement, and motivation. Similarly, early failures should be quickly addressed.

Specifically related again to free sugars, some nuance to the recommendations may also be required. We found some evidence that free sugars specifically were vilified by some participants in our study, with some increased reporting of rigid and unbalanced thinking in terms of restriction and reward. These concerns add further weight to the need for information on free sugar intakes in context, when considering diet as a whole, as part of a healthy lifestyle.

### Strengths and limitations

The strengths of this study lie in the large number of interviews undertaken, the random and inclusive nature of these interviews, and the collection of data by study group and time point. The depth and breadth of data gathered led to a substantial codebook, but this amount of data rendered the first summarised matrices for the framework analyses too unwieldly for analysis, resulting in further reduction of all data. This may have resulted in the loss of important and interesting differences between individuals. Furthermore, all interviews were carried out as part of a larger study, and the selective nature for enrolment into this study will have resulted in a select sample for this sub-study. Our findings thus may be specific to individuals volunteering for dietary change and may not be generalisable to individuals who do not volunteer for this, for example, those who are prescribed a dietary change for health reasons, or to the population in general. Some themes, for example, those around willingness and motivation may be particularly impacted by these concerns. The interviews were also only undertaken during the study, thus we have no perceptions following completion, and no interviews from individuals who withdrew. Perceptions may have changed following cessation of the study period, and drop-out from the study was low,^(18)^ but perceptions may certainly have differed in those who chose to leave the study rather than stay. Our study must also be considered in context – the work was undertaken in the UK, where the government recommends free sugar intakes at less than 5% TEI. While the WHO recommends free sugar intakes at less than 10% TEI with optimal intakes at less than 5% TEI, both recommendations aim to reduce free sugar consumption to reduce health risks. Our interviews and participants focussed on this broad aim rather than any specific goal. Our use of the 5% TEI goal may have resulted in some barriers to change that may not have been found in response to a lesser challenge, but all barriers will depend on the perception of the challenge to the individual undertaking it, rather than the absolute nature of the challenge. As a result, we consider our study and our results to be applicable to reducing free sugar intakes in general, and a range of other dietary challenges.

### Conclusions

This study aimed to identify barriers and facilitators to reducing free sugar intakes while participants attempted to do this as part of a randomised controlled trial and then analyse these barriers and facilitators based on intervention received, time for change, and degree of success at the end of a 12-week period. From interviews with 62 participants, we identified seven interactive themes leading to dietary change: ‘Is it possible?’; ‘Power of Knowledge’; ‘Personal Balance and Empowerment’; ‘Habitual Approach’; ‘Realities of Life’; ‘Extensive Awareness and Viewpoint’; and ‘Proof and Impact’. Differences were found in the content of these themes based on intervention received, time for change, and degree of success. By intervention, participants reported greater knowledge, with greater detail and discussion on practical solutions, in intervention groups compared to control. By time for change, participants reported intentions and expectations at the start of the process, followed by increasing or decreasing engagement and satisfaction over time, with a crucial time point at 4 weeks when physical and subjective proofs of changes started occurring. By degree of success at the end of 12 weeks, those with greater success reported more active engagement with the task, and growing knowledge, confidence, and motivation compared with those who were less successful. Based on the variation found, for reducing free sugar intakes, we recommend increasing knowledge, including knowledge specific to free sugar content; providing practical solutions which are small, manageable, and offer choice; ensuring expectations for change are realistic, with specific note of the need to engage for at least four weeks; supporting early successes and addressing early failures; and setting free sugar consumption in context encouraging moderation and balance to diet and lifestyle as a whole.

## Supporting information

10.1017/jns.2026.10118.sm001 Boxall et al. supplementary material Boxall et al. supplementary material

## Data Availability

Data described in the manuscript will be made available upon request.

## Supplementary material

The supplementary material for this article can be found at https://doi.org/10.1017/jns.2026.10118.

## Table 2. Long description

The table presents adherence scores ranging from zero to five across four groups: Control, N, NF, and NFS. It has six rows for each adherence score and five columns for the groups. Row 1: Zero, Control 2, N 2, NF 0, NFS 2. Row 2: One, Control 3, N 1, NF 1, NFS 2. Row 3: Two, Control 3, N 2, NF 3, NFS 4. Row 4: Three, Control 0, N 3, NF 2, NFS 7. Row 5: Four, Control 2, N 1, NF 3, NFS 3. Row 6: Five, Control 1, N 5, NF 7, NFS 6. The table highlights variations in adherence scores among the groups, with notable differences in higher adherence scores for the N, NF, and NFS groups compared to the Control group.

Navigate back to Table 2..

## Figure 1. Long description

The diagram illustrates seven interconnected themes from a thematic analysis. The themes are Power of Knowledge, Habitual Approach, Is It Possible, Personal Balance and Empowerment, Realities of Life, Extensive Awareness and Viewpoint, and Proof and Impact. Arrows indicate the relationships and flow between these themes, suggesting a cyclical process. The overall structure emphasizes the dynamic interactions among the themes, leading to making dietary changes.

Navigate back to Figure 1..

## Table 3. Long description

The table presents seven themes and fourteen subthemes related to barriers and facilitators to intervention adherence. Each theme is defined and accompanied by its subthemes where applicable. The themes include ‘Is it possible?’, ‘Power of knowledge’, ‘Personal balance and empowerment’, ‘Habitual approach’, ‘Realities of life’, ‘Extensive awareness and viewpoint’, and ‘Proof and impact’. Subthemes under ‘Power of knowledge’ are ‘Enables’ and ‘Disables’. Subthemes under ‘Personal balance and empowerment’ are ‘Balanced’, ‘Unbalanced’, ‘Empower’, and ‘Disempower’. Subthemes under ‘Habitual approach’ are ‘Active’ and ‘Passive’. Subthemes under ‘Realities of life’ are ‘Facilitators’ and ‘Barriers’. Subthemes under ‘Proof and impact’ are ‘Seeing proof’ and ‘Limited impact’. The table provides a structured overview of these themes and subthemes, highlighting their roles in intervention adherence.

Navigate back to Table 3..

## Table 4. Long description

A table with four rows and three columns comparing group commentary results across themes. The columns are labeled Theme, Similarities, and Differences. The table presents data from four groups: Control group, Group N, Group NF, and Group NFS. The Control group had 11 participants, Group N had 14, Group NF had 16, and Group NFS had 24. The table highlights that commentary increased across the groups, with the NFS group contributing the most. The Control group’s responses were more general and less personalized compared to the intervention groups. Themes include Is it possible, Power of knowledge, Personal balance and empowerment, Habitual approach, Realities of life, Extensive awareness and viewpoint, and Proof and impact. Notable trends include the importance of nutritional education, the influence of external observation, and the challenges of eating away from home.

Navigate back to Table 4..

## Table 5. Long description

The table presents a comparison of interview week results, highlighting similarities and differences across various time points. The interviews were conducted at week one with nine individuals, week two with eleven individuals, week four with thirteen individuals, week eight with fourteen individuals, and week twelve with eighteen individuals. The table is organized into themes such as ‘Is it possible?’, ‘Power of knowledge’, ‘Personal balance and empowerment’, ‘Habitual approach’, ‘Realities of life’, ‘Extensive awareness and viewpoint’, and ‘Proof and impact’. Each theme lists similarities and differences observed during the interviews. For instance, under ‘Is it possible?’, recommendations were generally described as achievable, but earlier interviews showed individuals trying to achieve guidelines, while later weeks indicated fatigue with dietary recording. The ‘Power of Knowledge’ theme noted individuals’ existing good knowledge but criticized food labeling as deceptive. ‘Personal balance and empowerment’ recognized personal responsibility and healthier choices, with later interviews showing increased awareness of bodily needs and the value of diet personalization. The ‘Habitual approach’ theme indicated motivation from observation or study participation, with later weeks showing more active changes in habits. ‘Realities of life’ discussed reducing portion sizes and lifestyle barriers, with later interviews highlighting friends and family as facilitators. ‘Extensive awareness and viewpoint’ showed increased thinking about changes and focus on labels and nutritional information over time. ‘Proof and impact’ noted the usefulness of dietary recording and physical changes observed from week one onwards.

Navigate back to Table 5..

## Table 6. Long description

A table with six rows and three columns compares adherence scores and themes, highlighting similarities and differences. The columns are labeled ‘Theme’, ‘Similarities’, and ‘Differences’. Themes include ‘Is it possible?’, ‘Power of Knowledge’, ‘Personal balance and empowerment’, ‘Habitual approach’, ‘Realities of life’, ‘Extensive awareness and viewpoint’, and ‘Proof and impact’. Each row details specific comments and adherence scores, ranging from lower to higher adherence, with notable trends in confidence, understanding, and empowerment.

Navigate back to Table 6..

## References

[1] Huang Y , Chen Z , Chen B , et al. Dietary sugar consumption and health: umbrella review. BMJ. 2023;381:e071609. 10.1136/bmj-2022-071609. 37019448 PMC10074550

[2] World Health Organization. Guideline: sugars intake for adults and children. 2015. Accessed June 10, 2026. https://www.who.int/publications/i/item/9789241549028. 25905159

[3] Walton J , Bell H , Re R , Nugent AP. Current perspectives on global sugar consumption: definitions, recommendations, population intakes, challenges and future direction. Nutr Res Rev. 2023;36:1–22. 10.1017/S095442242100024X. 34369326

[4] Public Health England. National Diet and Nutrition Survey Rolling programme Years 9 to 11 (2016/2017 to 2018/2019). 2020. Accessed June 10, 2026. https://assets.publishing.service.gov.uk/government/uploads/system/uploads/attachment_data/file/943114/NDNS_UK_Y9-11_report.pdf.

[5] Avery A , Bostock L , Mccullough F. A systematic review investigating interventions that can help reduce consumption of sugar-sweetened beverages in children leading to changes in body fatness. J Hum Nutr Diet. 2015;28(s1):52–64. 10.1111/jhn.12267. 25233843 PMC4309175

[6] Backholer K , Sarink D , Beauchamp A , et al. The impact of a tax on sugar-sweetened beverages according to socio-economic position: a systematic review of the evidence. Public Health Nutr. 2016;19(17):3070–3084. 10.1017/S136898001600104X. 27182835 PMC10270974

[7] Levy DT , Friend KB , Wang YC. A review of the literature on policies directed at the youth consumption of sugar sweetened beverages. Adv Nutr. 2011;2:182s–200s. 22332051 10.3945/an.111.000356 PMC3065753

[8] Vargas-Garcia EJ , Evans CEL , Prestwich A , Sykes-Muskett BJ , Hooson J , Cade JE. Interventions to reduce consumption of sugar-sweetened beverages or increase water intake: evidence from a systematic review and meta-analysis. Obes Rev. 2017;18:1350–1363. 10.1111/obr.12580. 28721697

[9] von Philipsborn P , Stratil JM , Burns J , et al. Environmental interventions to reduce the consumption of sugar-sweetened beverages and their effects on health. Cochrane Database Syst Rev. 2019;6:CD012292. 31194900 10.1002/14651858.CD012292.pub2 PMC6564085

[10] Appleton KM , van den Heuvel E. Chapter 37: how to create nutritional behaviour change. In: Wilson T , Temple NJ , Bray GA , eds. Nutritional Science for Physicians and Related Health Care Professionals, (3rd ed.). Springer Nature Humana Press; 2022. 10.1007/978-3-030-82515-7.

[11] Deslippe AL , Soanes A , Bouchaud CC , et al. Barriers and facilitators to diet, physical activity and lifestyle behavior intervention adherence: a qualitative systematic review of the literature. Int J Behav Nutr Phy. 2023;20(1):14. 10.1186/S12966-023-01424-2. PMC9925368 36782207

[12] Gupta A , Smithers LG , Harford J , Merlin T , Braunack-Mayer A. Determinants of knowledge and attitudes about sugar and the association of knowledge and attitudes with sugar intake among adults: a systematic review. Appetite. 2018;126:185–194. 10.1016/J.APPET.2018.03.019. 29634988

[13] Mazarello Paes V , Hesketh K , O.’Malley C , et al. Determinants of sugar-sweetened beverage consumption in young children: a systematic review. Obes Rev. 2015;16(11):903–913. 10.1111/obr.12310. 26252417 PMC4737242

[14] Rawahi SH , Asimakopoulou K , Newton JT. Factors related to reducing free sugar intake among white ethnic adults in the UK: a qualitative study. BDJ Open. 2018;4(1):1–6. 10.1038/bdjopen.2017.24. PMC5842859 29607093

[15] Tang CS , Mars M , James J , Appleton KM. Associations between attitudes towards and reported intakes of sugars, low/no-calorie sweeteners and sweet-tasting foods in a UK sample. Appetite. 2024;194:107169. 10.1016/j.appet.2023.107169. 38113982

[16] Tang CS , Mars M , James J , de Graaf K , Appleton KM. Sweet talk: a qualitative study exploring attitudes towards sugars, sweeteners and sweet-tasting foods in the United Kingdom. Foods. 2021;10:1172. 10.3390/foods10061172. 34073676 PMC8225159

[17] Boxall LR , Arden-Close E , James J , Appleton KM. Protocol: the effects of nutrient-vs food-vs food-substitution-based dietary recommendations for reducing free sugar intakes, on free sugar intakes, dietary profiles and sweet taste outcomes: a randomised controlled trial. Nutr Health. 2024;30:269–278. 10.1177/02601060221111234. 35818972 PMC11141080

[18] Boxall LR , Arden-Close E , James J , Appleton KM. Effects of dietary recommendations for reducing free sugar intakes, on free sugar intakes, dietary profiles and anthropometry: a randomised controlled trial. Brit J Nutr. 2025;133:694–710. 10.1017/S0007114525000339. 39973355 PMC12055439

[19] Suresh K. An overview of randomization techniques: an unbiased assessment of outcome in clinical research. J Hum Reprod Sci. 2011;4:8–11. 10.4103/0974-1208.82352. 21772732 PMC3136079

[20] Patton MQ. Qualitative Research & Evaluation Methods (4th ed.). SAGE Publications, Inc; 2014. https://us.sagepub.com/en-us/nam/qualitative-research-evaluation-methods/book232962.

[21] Brayda WC , Boyce TD. So you really want to interview me?: navigating “Sensitive” qualitative research interviewing. Int J Qual Meth. 2014;13:318–334. 10.1177/160940691401300115.

[22] Smith J , Firth J. Qualitative data analysis: application of the framework approach. Nurse Res. 2011;18(2):52–62. 21319484 10.7748/nr2011.01.18.2.52.c8284

[23] Braun V , Clarke V. Successful Qualitative Research: A Practical Guide for Beginners. Sage; 2013.

[24] Gale NK , Heath G , Cameron E , Rashid S , Redwood S. Using the framework method for the analysis of qualitative data in multi-disciplinary health research. BMC Med Res Methodol. 2013;13:117. 10.1186/1471-2288-13-117. 24047204 PMC3848812

[25] Goldsmith LJ. Using framework analysis in applied qualitative research. Qual Rep. 2021;26(6):2061–2076. 10.46743/2160-3715/2021.5011.

[26] Munt AE , Partridge SR , Allman-Farinelli M. The barriers and enablers of healthy eating among young adults: a missing piece of the obesity puzzle: a scoping review. Obes Rev. 2017;18:1–17. 10.1111/obr.12472. 27764897

[27] Silva P , Araújo R , Lopes F , Ray S. Nutrition and food literacy: framing the challenges to health communication. Nutrients. 2023;15:4708. 10.3390/NU15224708. 38004102 PMC10674981

[28] Tsofliou F , Vlachos D , Hughes C , Appleton KM. Barriers and facilitators associated with the adoption of and adherence to a Mediterranean style diet in adults: a systematic review of published observational and qualitative studies. Nutrients. 2022;14:4314. 10.3390/nu14204314. 36296998 PMC9607475

[29] López-Hernández L , Martínez-Arnau FM , Pérez-Ros P , Drehmer E , Pablos A. Improved nutritional knowledge in the obese adult population modifies eating habits and serum and anthropometric markers. Nutrients. 2020;12:3355. 10.3390/NU12113355. 33143306 PMC7693073

[30] Petrovici DA , Ritson C. Factors influencing consumer dietary health preventative behaviours. BMC Public Health. 2006;6:222. 10.1186/1471-2458-6-222. 16948839 PMC1584406

[31] Rustad C , Smith C. Nutrition knowledge and associated behavior changes in a holistic, short-term nutrition education intervention with low-income women. J Nutr Educ Behav. 2013;45:490–498. 10.1016/j.jneb.2013.06.009. 24206584

[32] Hattersley L , Irwin M , King L , Allman-Farinelli M. Determinants and patterns of soft drink consumption in young adults: a qualitative analysis. Public Health Nutr. 2009;12:1816–1822. 10.1017/s136898000800462x. 19195421

[33] Huffman L , West DS. Readiness to change sugar sweetened beverage intake among college students. Eat Behav. 2007;8:10–14. 17174846 10.1016/j.eatbeh.2006.04.005

[34] Bandura A. Self-efficacy: toward a unifying theory of behavioral change. Psychol Rev. 1977;84:191–215. 10.1037/0033-295X.84.2.191. 847061

[35] Bandura A. Health promotion by social cognitive means. Health Educ Behav. 2004;31:143–164. 10.1177/1090198104263660. 15090118

[36] Barbosa HC , de Queiroz Oliveira JA , da Costa JM , et al. Empowerment-oriented strategies to identify behavior change in patients with chronic diseases: an integrative review of the literature. Patient Educ Couns. 2021;104:689–702. 10.1016/j.pec.2021.01.011. 33478854

[37] Buckworth J. Promoting self-efficacy for healthy behaviors. ACSM Health Fit J. 2017;21:40–42. 10.1249/FIT.0000000000000318.

[38] Lindvall K , Larsson C , Weinehall L , et al. Weight maintenance as a tight rope walk - a Grounded Theory study. BMC Public Health. 2010;10:51. 10.1186/1471-2458-10-51. 20122140 PMC2835685

[39] OLeary A. Self-efficacy and health. Behav Res Ther. 1985;23(4):437–451. 10.1016/0005-7967(85)90172-X. 3896228

[40] Robinson K , Muir S , Newbury A , Santos-Merx L , Appleton KM. Perceptions of body-weight that vary by Body Mass Index: clear associations with perceptions based on personal control and responsibility. J Health Psychol. 2022;27:147–165. 10.1177/1359105320916540. 32431165 PMC8739579

[41] Strecher VJ , McEvoy DeVellis B , Becker MH , Rosenstock IM. The role of self-efficacy in achieving health behavior change. Health Educ Quart. 1986;13(1):73–92. 10.1177/109019818601300108. 3957687

[42] Welsh EM , Jeffery RW , Levy RL , et al. Measuring perceived barriers to healthful eating in obese, treatment-seeking adults. J Nutr Educ Behav. 2012;44:507–512. 10.1016/j.jneb.2010.06.005. 21665549 PMC3175339

[43] Atanasova P , Kusuma D , Pineda E , Frost G , Sassi F , Miraldo M. The impact of the consumer and neighbourhood food environment on dietary intake and obesity-related outcomes: a systematic review of causal impact studies. Soc Sci Med. 2022;299:114879. 10.1016/j.socscimed.2022.114879. 35290815 PMC8987734

[44] Herforth A , Ahmed S. The food environment, its effects on dietary consumption, and potential for measurement within agriculture-nutrition interventions. Food Secur. 2015;7:505–520. 10.1007/s12571-015-0455-8.

[45] Popkin BM , Duffey K , Gordon-Larsen P. Environmental influences on food choice, physical activity and energy balance. Physiol Behav. 2005;86(5):603–613. 10.1016/J.PHYSBEH.2005.08.051. 16246381

[46] Bowen DJ , Barrington WE , Beresford SAA. Identifying the effects of environmental and policy change interventions on healthy eating. Annu Rev Publ Health. 2015;36:289. 10.1146/ANNUREV-PUBLHEALTH-032013-182516. PMC4583099 25785891

[47] Vaughan CA , Collins R , Ghosh-Dastidar M , Beckman R , Dubowitz T. Does where you shop or who you are predict what you eat?: the role of stores and individual characteristics in dietary intake. Prev Med. 2017;100:10–16. 10.1016/J.YPMED.2017.03.015. 28341459 PMC5480899

[48] Chung LMY , Law QPS , Fong SSM , Chung JWY. Electronic dietary recording system improves nutrition knowledge, eating attitudes and habitual physical activity: a randomised controlled trial. Eat Behav. 2014;15:410–413. 10.1016/J.EATBEH.2014.04.011. 25064291

[49] Elfhag K , Rössner S. Who succeeds in maintaining weight loss? A conceptual review of factors associated with weight loss maintenance and weight regain. Obes Rev. 2005;6:67–85. 10.1111/j.1467-789X.2005.00170.x. 15655039

[50] Ingels JS , Misra R , Stewart J , Lucke-Wold B , Shawley-Brzoska S. The effect of adherence to dietary tracking on weight loss: using HLM to model weight loss over time. J Diabetes Res. 2017;2017:6951495. 10.1155/2017/6951495. 28852651 PMC5568610

[51] Hollis JF , Gullion CM , Stevens VJ , et al. Weight loss during the intensive intervention phase of the weight-loss maintenance trial. Am J Prev Med. 2008;35:118–126. 10.1016/J.AMEPRE.2008.04.013. 18617080 PMC2515566

[52] Bazrafkan L , Choobineh MA , Shojaei M , et al. How do overweight people dropout of a weight loss diet? A qualitative study. BMC Nutr. 2021;7:76. 10.1186/s40795-021-00480-w. 34794513 PMC8603507

[53] Colombo O , Ferretti VV , Ferraris C , et al. Is drop-out from obesity treatment a predictable and preventable event? Nutr J. 2014;13:13. 10.1186/1475-2891-13-13. 24490952 PMC3914843

[54] Goode RW , Ye L , Sereika SM , et al. Socio-demographic, anthropometric, and psychosocial predictors of attrition across behavioral weight-loss trials. Eat Behav. 2016;20:27–33. 10.1016/j.eatbeh.2015.11.009. 26609668 PMC4826274

[55] James A , Lawrence B , O’Connor M. Healthy eating as a new way of life: a qualitative study of successful long-term diet change. Inquiry. 2022;59:00469580221090397. 10.1177/00469580221090397. 35418258 PMC9016560

[56] Perna S , Spadaccini D , Riva A , et al. A path model analysis on predictors of dropout (at 6 and 12 months) during the weight loss interventions in endocrinology outpatient division. Endocrine. 2018;61:447–461. 10.1007/s12020-018-1563-y. 29470776

[57] Teixeira PJ , Silva MN , Mata J , Palmeira AL , Markland D. Motivation, self-determination, and long-term weight control. Int J Behav Nutr Phy. 2012;9(1):1–13. 10.1186/1479-5868-9-22. PMC3312817 22385818

[58] Deci EL , Ryan RM. Intrinsic Motivation and Self-Determination in Human Behavior. Plenum Press; 1985.

[59] Deci EL , Ryan RM. The ‘what’ and ‘why’ of goal pursuits: human needs and the self-determination of behavior. Psychol Inq. 2000;11:227–268.

[60] Hagger MS , Hardcastle SJ , Chater A , et al. Autonomous and controlled motivational regulations for multiple health-related behaviors: between-and within-participants analyses. Health Psychol Behav Med. 2014;2:565–601. 10.1080/21642850.2014.912945. 25750803 PMC4346087

[61] Ng JY , Ntoumanis N , Thøgersen-Ntoumani C , et al. Self-determination theory applied to health contexts: a meta-analysis. Perspect Psychol Sci. 2012;7:325–340. 10.1177/1745691612447309. 26168470

[62] Hill JO. Can a small-changes approach help address the obesity epidemic? A report of the joint task force of the American society for nutrition, institute of food technologists, and international food information council. Am J Clin Nutr. 2009;89:477–484. 19088151 10.3945/ajcn.2008.26566

[63] Hills AP , Byrne NM , Lindstrom R , Hill JO. Small changes’ to diet and physical activity behaviors for weight management. Obes Facts. 2013;6:228–238. 10.1159/000345030. 23711772 PMC5644785

[64] Rodearmel SJ , Wyatt HR , Barry MJ , et al. A family-based approach to preventing excessive weight gain. Obesity. 2006;14:1392–1401. 10.1038/oby.2006.158. 16988082

[65] Rodearmel SJ , Wyatt HR , Stroebele N , Smith S , Ogden L , Hill JO. Small changes in dietary sugar and physical activity as an approach to preventing excessive weight gain: the America on the Move Family Study. Pediatrics. 2007;120:869–879. 10.1542/peds.2006-2927 17908743

[66] Stroebele N , de Castro JM , Stuht J , Catenacci V , Wyatt HR , Hill JO. A small-changes approach reduces energy intake in free-living humans. J Am Coll Nutr. 2009;28:63–68. 10.1080/07315724.2009. 19571162 PMC2894414

[67] Bailey RR. Goal setting and action planning for health behavior change. Am J Lifestyle Med. 2017;13(6):615. 10.1177/1559827617729634. 31662729 PMC6796229

[68] Locke EA , Latham GP. Building a practically useful theory of goal setting and task motivation: a 35-year odyssey. Am Psychol. 2002;57:705. 10.1037/0003-066X.57.9.705. 12237980

[69] Locke EA , Latham GP. New directions in goal-setting theory. Curr Dir Psychol Sci. 2006;15:265–268. 10.1111/j.1467-8721.2006.00449.x.

[70] Ebbeling CB , Feldman HA , Steltz SK , Quinn NL , Robinson LM , Ludwig DS. Effects of sugar-sweetened, artificially sweetened, and unsweetened beverages on cardiometabolic risk factors, body composition, and sweet taste preference: a randomized controlled trial. J Am Heart Assoc. 2020;9:e015668. 10.1161/JAHA.119.015668. 32696704 PMC7792240

[71] Tate DF , Turner-McGrievy G , Lyons E , et al. Replacing caloric beverages with water or diet beverages for weight loss in adults: main results of the Choose Healthy Options Consciously Everyday (CHOICE) randomized clinical trial. Am J Clin Nutr. 2012;95:555–563. 10.3945/ajcn.111.026278. 22301929 PMC3632875

[72] Wise PM , Nattress L , Flammer LJ , Beauchamp GK. Reduced dietary intake of simple sugars alters perceived sweet taste intensity but not perceived pleasantness. Am J Clin Nutr. 2016;103:50–60. 10.3945/ajcn.115.112300. 26607941

[73] Herforth A , Arimond M , Álvarez-Sánchez C , Coates J , Christianson K , Muehlhoff E. A global review of food-based dietary guidelines. Adv Nutr. 2019;10:590–605. 10.1093/advances/nmy130. 31041447 PMC6628851

[74] Kwasnicka D , Dombrowski SU , White M , Sniehotta F. Theoretical explanations for maintenance of behaviour change: a systematic review of behaviour theories. Health Psychol Rev. 2016;10:277–296. 10.1080/17437199.2016.1151372. 26854092 PMC4975085

[75] Bielat AD , Rogers PJ , Appleton KM. Effects of a six-day, whole-diet sweet taste intervention on pleasantness, desire for, and intakes of sweet foods: a randomised controlled trial. Brit J Nutr. 2025;133:277–288. 10.1017/S0007114524003209. 39698772 PMC11813625

[76] Miller LM , Cassady DL. The effects of nutrition knowledge on food label use. A review of the literature. Appetite. 2015;92:207–216. 10.1016/j.appet.2015.05.029. 26025086 PMC4499482

[77] Department of Health. Guide to creating a front of pack (FoP) nutrition label for pre-packed products sold through retail outlets. 2016. Accessed June 10, 2026. https://www.food.gov.uk/sites/default/files/media/document/fop-guidance_0.pdf.

[78] Department of Health. Technical guidance on nutrition labelling. 2016. Accessed June 10, 2026. https://assets.publishing.service.gov.uk/media/5a8010d8e5274a2e87db7a62/Nutrition_Technical_Guidance.pdf.

[79] Krukowski RA , Denton AH , König LM. Impact of feedback generation and presentation on self-monitoring behaviors, dietary intake, physical activity, and weight: a systematic review and meta-analysis. Int J Behav Nutr Phy. 2024;21:1–18. 10.1186/S12966-023-01555-6/TABLES/4. PMC10765525 38178230

[80] Chung LMY , Fong SSM. Role of behavioural feedback in nutrition education for enhancing nutrition knowledge and improving nutritional behaviour among adolescents. Asia Pac J Clin Nutr. 2018;27:466–472. 10.6133/apjcn.042017.03. 29384337

[81] Hooson J , Hutchinson J , Warthon-Medina M , et al. A systematic review of reviews identifying UK validated dietary assessment tools for inclusion on an interactive guided website for researchers: www.nutritools.org. Crit Rev Food Sci. 2020;60:1265. 10.1080/10408398.2019.1566207. PMC7114915 30882230

[82] Trabulsi J , Schoeller DA. Evaluation of dietary assessment instruments against doubly labeled water, a biomarker of habitual energy intake. Am J Physiol Endocrinol Metab. 2001;281:E891–E899. 10.1152/ajpendo.2001.281.5.E891. 11595643

[83] Hahn SL , Kaciroti N , Eisenberg D , Weeks HM , Bauer KW , Sonneville KR. Introducing dietary self-monitoring to undergraduate women via a calorie counting app has no effect on mental health or health behaviors: results from a randomized controlled trial. J Acad Nutr Diet. 2021;121:2377–2388. 10.1016/j.jand.2021.06.311. 34427188 PMC9109125

[84] Jospe MR , Brown RC , Williams SM , Roy M , Meredith-Jones KA , Taylor RW. Self-monitoring has no adverse effect on disordered eating in adults seeking treatment for obesity. Obes Sci Pract. 2018;4:283–288. 10.1002/osp4.168. 29951219 PMC6010018

[85] Ellis S , Rosenblum K , Miller A , et al. Meaning of the terms ‘overweight’ and ‘obese’ among low-income women. J Nutr Educ Behav. 2014;46:299–303. 10.1016/j.jneb.2013.08.006. 24135314 PMC3986347

[86] Puhl RM , Brownell KD. Psychological origins of obesity stigma: toward changing a powerful and pervasive bias. Obes Rev. 2003;4:213–227. 10.1046/j.1467-789x.2003.00122.x. 14649372

