# Supplementary material for: Barriers and facilitators to dietary change while aiming to reduce free sugar intakes: framework analysis based on intervention, time for change, and success at 12 weeks
Source: J Nutr Sci. 2026 Jun 18;15:e47. doi: 10.1017/jns.2026.10118 (PMC13279952; doi:10.1017/jns.2026.10118)
Supplement: Boxall et al. supplementary material [file S2048679026101189sup001.docx]

**Barriers and facilitators to dietary change while aiming to reduce free sugar intakes: Framework analysis based on intervention, time for change and success at 12 weeks**

**Lucy R Boxall, Hannah Dalby, Emily Arden-Close, Janet James, and Katherine M Appleton.**

## **Supplementary materials**

**S1: Semi-Structured participant interview script**

Questions aimed at identifying each individuals’ barriers and facilitators that affect their adherence to dietary recommendations. ___________________________________________________________________________

**Introduction**

In general, how do you feel about dietary recommendations as a whole?

Anything positive/negative **(Prompt)**

Are you currently following the recommendations you were given?

Were you ever following them?

**Knowledge/ behaviour/ experience**

1. What was your first thought on receiving your dietary recommendation?
2. How do you feel about your progress so far?
3. Are there any elements of the recommendations in particular that you enjoy?
4. Have you found any difficulties?
5. Would anything have helped?
   - If so what? **(Prompt)**
6. Can you describe any recent changes to your eating behaviours/ habits since starting the study?
   - Changes to the types of foods you eat? **(Prompt)**
   - The number of times you eat? **(Prompt)**
   - The pleasure? **(Prompt)**
   - Taste? **(Prompt)**
7. Are there any dietary changes you would have liked to make but have been unable to?
   - If yes, then why?
8. Can you describe if following the recommendations has affected your life?
   - If so how? **(Prompt)**
9. Has it affected the lives of those around you?
10. Has your view on the relationship between diet and health changed since starting the study?
11. Have the dietary recommendations affected your food/dietary knowledge?
    - If so how? **(Prompt)**
12. Can you think of anyway the recommendations may be improved?

**Opinions and beliefs**

1. Thinking about the guidelines you received, How achievable do you think the dietary recommendations are?
   - For yourself/individual? **(Prompt)**
   - And for the general public **(Prompt)**
2. What could be done to make achieving these goals easier?
3. Do family & friends have any influence in achieving these goals?
   - Does the government have a role? **(Prompt)**
   - The food industry? **(Prompt)**
   - Or is it all up to the individual and their free choice? **(Prompt)**

**Background/demographic**

1. Which (if any) factors influence your diet that are out of your control?
2. Is there anything unique to you which you think may affect your ability to achieve these dietary goals?
3. What advice would you give to others following the same recommendations?

**Conclusion**

1. Thank you for taking the time to participate in this interview, is there anything you would like to add before we end?

**END OF INTERVIEW**
